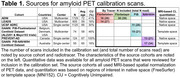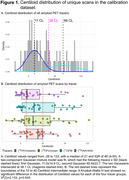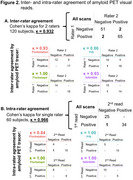# Calibration of multi‐site raters for prospective visual read of amyloid PET scans acquired across the ADRC Consortium for Clarity in ADRD Research Through Imaging (CLARiTI)

**DOI:** 10.1002/alz70856_107616

**Published:** 2026-01-09

**Authors:** David N. Soleimani‐Meigooni, Ganna Blazhenets, Renaud La Joie, Zoe Lin, Carol Soppe, Derek R. Johnson, Mary Ellen I. Koran, Jonathan E. McConathy, Ilya M. Nasrallah, Jeremy A. Tanner, Victor L. Villemagne, Charles C. Windon, Michael Zeineh, Elizabeth C. Mormino, Sterling C Johnson, Gil D. Rabinovici

**Affiliations:** ^1^ Memory and Aging Center, Weill Institute for Neurosciences, University of California San Francisco, San Francisco, CA, USA; ^2^ Lawrence Berkeley National Laboratory, Berkeley, CA, USA; ^3^ Department of Radiology, Mayo Clinic, Rochester, MN, USA; ^4^ Department of Radiology, Mayo Clinic, Phoenix, AZ, USA; ^5^ University of Alabama at Birmingham, Birmingham, AL, USA; ^6^ University of Pennsylvania, Philadelphia, PA, USA; ^7^ Glenn Biggs Institute for Alzheimer's & Neurodegenerative Diseases, University of Texas Health Science Center, San Antonio, TX, USA; ^8^ University of Pittsburgh School of Medicine, Pittsburgh, PA, USA; ^9^ Stanford University School of Medicine, Stanford, CA, USA; ^10^ Wisconsin Alzheimer's Institute, University of Wisconsin‐Madison School of Medicine and Public Health, Madison, WI, USA; ^11^ Department of Radiology and Biomedical Imaging, University of California San Francisco, San Francisco, CA, USA

## Abstract

**Background:**

PET visual reads for multi‐center studies are conducted centrally by one or a few experts. In CLARiTI, a broader network of raters will perform the reads, necessitating methods to ensure their accuracy and reliability.

**Method:**

All ADRC sites (*N* = 37) will prospectively acquire amyloid PET scans for 800 cognitively unimpaired and 1200 cognitively impaired participants. Each site can use one of four amyloid PET tracers ([^18^F]florbetaben, [^18^F]florbetapir, [^11^C]PIB, [^18^F]NAV4694). PET scans will be visually interpreted by ten PET neuroimaging experts located across eight ADRCs, using their preferred hardware, software, and file format (DICOM, NIFTI). Calibration requires readers to perform blinded, independent visual interpretation of 180 amyloid PET scans (30 unique and 15 duplicate scans per tracer), previously read and selected by an expert (Table 1). An even mix of visually non‐elevated and elevated scans were selected, with quantitation spanning ‐26‐123 Centiloids (Figure 1), and 15% were visually borderline (similar proportion to ADNI4), with quantitation spanning 4‐48 Centiloids (mean 21). Kruskal‐Wallis *H* test showed no significant difference in the distribution of Centiloid values for scans in each tracer group, Χ^2^(3)=2.133, *p* = 0.545.

**Result:**

Calibration scans were read by a second expert not serving in CLARiTI. There was 97% concordance and almost perfect agreement between readers (κ=0.932, *p* <0.001; Figure 2). Discordantly read scans were visually borderline elevated (*N* = 2), borderline non‐elevated (*N* = 1), and negative (*N* = 1), with quantitation of 8‐21 Centiloids (mean 14). Intra‐rater reads were 98% concordant and had almost perfect agreement (κ=0.966, *p* <0.001). These findings hold promise for scaling‐up visual reads in CLARiTI, and we expect ≥80% concordance and substantial to almost perfect agreement (κ≥0.7) for inter/intra‐rater reliability.

**Conclusion:**

Calibration is essential for accurate/reliable amyloid PET visual reads in large multi‐center studies with multiple raters. Through the calibration process, we will develop methods to improve the adjudication of borderline/challenging scans and formalize methods for visual reads of amyloid PET tracers that are investigational (i.e., [^11^C]PIB, [^18^F]NAV4694). Involving multiple raters across different sites, who use diverse hardware/software/file format combinations, will mirror the natural heterogeneity present in real‐world clinical practice. This calibration set can also serve as a standardized training tool for other studies incorporating amyloid PET visual reads.